# Sonoporation Delivery of Monoclonal Antibodies against Human Papillomavirus 16 E6 Restores p53 Expression in Transformed Cervical Keratinocytes

**DOI:** 10.1371/journal.pone.0050730

**Published:** 2012-11-30

**Authors:** Melissa Togtema, Samuel Pichardo, Robert Jackson, Paul F. Lambert, Laura Curiel, Ingeborg Zehbe

**Affiliations:** 1 Probe Development and Biomarker Exploration, Thunder Bay Regional Research Institute, Thunder Bay, Ontario, Canada; 2 Department of Biology, Lakehead University, Thunder Bay, Ontario, Canada; 3 Imaging Guided Interventions, Thunder Bay Regional Research Institute, Thunder Bay, Ontario, Canada; 4 McArdle Laboratory for Cancer Research, University of Wisconsin-Madison, Madison, Wisconsin, United States of America; Ospedale Pediatrico Bambino Gesu’, Italy

## Abstract

High-risk types of human papillomavirus (HPV), such as HPV16, have been found in nearly all cases of cervical cancer. Therapies targeted at blocking the HPV16 E6 protein and its deleterious effects on the tumour suppressor pathways of the cell can reverse the malignant phenotype of affected keratinocytes while sparing uninfected cells. Through a strong interdisciplinary collaboration between engineering and biology, a novel, non-invasive intracellular delivery method for the HPV16 E6 antibody, F127-6G6, was developed. The method employs high intensity focused ultrasound (HIFU) in combination with microbubbles, in a process known as sonoporation. In this proof of principle study, it was first demonstrated that sonoporation antibody delivery into the HPV16 positive cervical carcinoma derived cell lines CaSki and SiHa was possible, using chemical transfection as a baseline for comparison. Delivery of the E6 antibody using sonoporation significantly restored p53 expression in these cells, indicating the antibody is able to enter the cells and remains active. This delivery method is targeted, non-cytotoxic, and non-invasive, making it more easily translatable for *in vivo* experiments than other transfection methods.

## Introduction

Virtually all cervical cancers are dependent on persistent infection by high-risk human papillomavirus (HPV) [1]. Papillomaviruses are also implicated in almost 90% of other anogenital cancers [2]. In addition, oral cancer and non-melanoma skin cancer have an etiological association with high-risk HPVs [3].

Reliable screening procedures exist for cervical cancer, notably the Pap smear. However, cervical cancer still remains prevalent, particularly in populations with reduced access to screening, due to geographical or cultural limitations [4]. Cervical cancer commonly affects women in their thirties and forties [4], significantly impacting the quality of life during their active, younger years. The current treatment for cervical cancer, consisting of cisplatin/radiotherapy combined with surgery, has remained unchanged for the past several years despite its many detrimental side effects, including nausea, fatigue, and toxicity in unaffected organs. In addition, surgical excision of cervical cancerous tissue is a highly invasive procedure, and thus impractical. A more targeted therapy for cervical cancer would help decrease treatment-associated morbidity and overall mortality, and can also be applied to other HPV-related cancers, such as head and neck cancers, the incidence of which is currently on the rise [5].

HPV16 is the most common high-risk papillomavirus type, and like other tumourigenic DNA viruses, encodes viral oncoproteins that act synergistically [6]. Two intracellular oncoproteins, E6 and E7, play an important role in the malignant transformation of HPV-infected cells [6]. E7 induces increased cellular proliferation by binding to and inactivating the tumour suppressor retinoblastoma protein, thereby releasing a transcription factor (E2F) and allowing the HPV-infected cell to proceed through the cell cycle, even in the absence of growth factors [7]. E6 is the main player in cellular immortalization and transformation as well as in upholding tumour growth [8]. These activities are mediated by E6-dependent degradation of cellular proteins (reviewed in [9]) such as the tumour suppressor protein p53 [10] and by promoting telomerase activity [11].

Since E6 is crucial for cervical carcinogenesis and most importantly for maintenance of the malignant phenotype [12], [13], this molecule is an attractive target for new treatment strategies. Initially, small molecule approaches were tried. A library screen of small molecules identified zinc-finger ejecting compounds targeting E6 [14], [15]. However, these compounds have not had the anticipated effect [16] or required excessively high doses to be clinically relevant [15]. Thus, the rational design of small molecules as therapeutic agents that target specific proteins is extremely challenging due to the complex energetics associated with small molecule-protein interactions. Using large molecules has been more successful: therapeutic anti-E6 gene product approaches, including ribozymes, siRNA, and antibodies have been highly effective in cell culture and animal models [17]–[21].

Anti-E6, large molecule therapeutics require crossing cell membranes to be effective against HPV-induced cancers. Chemical transfection reagents are an easy solution to this problem *in vitro*, but the cytotoxic nature of these reagents and their potential to cause unwanted immunogenic effects limits their use *in vivo* and in clinical environments. A variety of other methods to facilitate cell membrane crossing, including the use of membrane translocating signal transport peptides, electroporation, and even red cell ghosts [22]–[24], have been explored, but again lack ease of translation.

Ideally, localized excitation of the membrane that results in transient increased permeability would be well-suited for a clinical application. Such an excitation can be produced by ultrasound, and indeed, high intensity focused ultrasound (HIFU) combined with microbubbles (lipid shell-encased octafluoropropane gas contrast agents), a process known as sonoporation, has been used for ultrasound-mediated intracellular delivery of a variety of molecules such as dextrans, calcein, plasmid DNA, siRNA, and antibodies (Table 1) [25]–[34]. Mechanistic studies have implied plasma membrane sonoporation as the dominant mechanism underlying ultrasound-enhanced molecule transfer [35]. Reversible pore formation, approximately 100 nm in effective diameter with a half-life of a few seconds, is thought to result from mechanical stress to the cell membrane caused by oscillation and cavitation of the microbubbles under the influence of the acoustic beam [35]. The formation of these pores has been studied using techniques such as: atomic force microscopy; high-speed camera, real-time optical observations of cell/bubble interactions; scanning electron microscopy; and measurement of changes in trans-membrane current [31], [36]–[38]. Microbubbles are routinely used today as an intravenously injected diagnostic drug for contrast enhancement during echocardiographic procedures.

**Table 1:** Examples of experiments using sonoporation to transfer large molecules across the cell membrane.

Group	Samples	Transfected Molecule	Microbubbles Used
Bao *et al*. 1997 [25]	Chinese Hamster Ovarian Cells	Luciferase plasmid, FITC-dextran	Albunex®
Forbes *et al*. 2008 [26]	Chinese Hamster Ovarian Cells	FITC-dextran	Optison®
Karshafian *et al*. 2010 [27]	KHT-C cells	FITC-dextran	Optison®, Definity®
Kinoshita & Hynynen 2007 [28]	C166 cells, C166-GFP cells	calcein, egfp siRNA	Optison®
Kinoshita & Hynynen 2005 [29]	HeLa cells, BJAB cells	calcein, Bak BH3 peptide	Optison®
Kinoshita & Hynynen 2005 [30]	BJAB cells, C166 cells, C166-GFP cells	calcein, egfp siRNA, negative control siRNA, pEGFP-C3	Optison®
Mehier-Humbert *et al*. 2005 [31]	MAT B III cells	FITC-dextran, fluorescent latex nanospheres(25, 44, 75 nm)	soft (1A009) and hard (BG1766) shelled ultrasound contrast agents
Meijering *et al*. 2009 [32]	primary bovine aortic endothelial cells,rat femoral arteries	tetramethylrhodamine isothiocyanate-dextran, FITC-dextran, lysine-fixable FITC-dextran	Sonovue®
Wu *et al*. 2006 [33]	Jurkat lymphocytes, human peripheral blood mononuclear cells	anti-rabbit IgG-Alexa Fluor® 647, anti-mouseIgD-FITC, Adriamycin hydrochloride	Optison®
Yudina *et al*. 2011 [34]	C6 cells	Sytox Green, Sytox Blue, TOTO-3 (intercalating fluorophores)	Sonovue®

The rationale of this work was to biologically verify sonoporation delivery of an anti-E6 antibody, using chemical transfection as a baseline for comparison and initial antibody characterization. This study demonstrated the effective delivery of a monoclonal antibody against HPV16 E6 using microbubble-mediated HIFU sonoporation, as evidenced by the resulting decrease in p53 degradation. The unique benefit of this novel approach is that, unlike other transfection methods (i.e. chemical), it is easily transferable to *in vivo* protocols, and potentially, even clinical trial-based experiments, thus filling the gap in translational research that these other methods were unable to address. The feasibility of monoclonal antibody delivery by sonoporation in cervical carcinoma cell lines was first evaluated by experiments using an antibody against the house-keeping gene product, tubulin. Preliminary chemical transfection experiments were then done to establish the biological effects a new E6 antibody (F127-6G6, not used before in this context), as well as the previously developed, well-described E6 antibody (4C6) had on p53 expression. Based on these results, primary sonoporation experiments were then carried out using the F127-6G6 E6 antibody.

## Materials and Methods

### Cell Culture

The cervical cancer-derived cell lines CaSki and SiHa (ATCC, Manassas, VA, USA) were maintained at 37°C, 5% CO_2_ in Dulbecco’s Modified Eagle Medium (DMEM; Sigma-Aldrich, Oakville, ON, Canada). The negative control, near-diploid immortalized keratinocytes (NIKS) [39] were grown in medium including Ham’s F12-medium and DMEM (3∶1), with 1% each of hydrocortisone, cholera toxin, insulin, adenine, and epidermal growth factor [40], [41]. Both types of media were supplemented with 10% and 2.5%, respectively, heat-inactivated fetal bovine serum (FBS; HyClone, Logan, UT, USA), 100 U of penicillin, 100 µg of streptomycin and 0.25 µg amphotericin B per mL (antibiotic/antimycotic; Gibco, Grand Island, NY, USA). Cells were passaged to sustain a 60% to 80% confluent monolayer and were routinely screened for *Mycoplasma* contamination.

### Monoclonal Antibodies

Mouse monoclonal anti-human antibodies (mAb) against tubulin (T6074; Sigma-Aldrich) and HPV16 E6 proteins, (F127-6G6 and 4C6; kind gifts of Arbor Vita Corporation, Fremont, CA, USA) were transfected into the cells both chemically and via sonoporation.

### HIFU Equipment and Contrast Agents

This study utilized the clinically approved ultrasound contrast agent, DEFINITY® perflutren [gaseous octafluoropropane (C_3_F_8_)] lipid microspheres (Lantheus Medical Imaging, North Billerica, MA, USA) in combination with HIFU to induce transient cell membrane permeability. The DEFINITY® microbubbles were activated before use by agitation for 45 s in a Vial-Mix (Lantheus Medical Imaging). After activation, 33 µL microbubbles were added to 10 mL of cell medium, with a final volume concentration of 0.33%.

**Figure 1:**
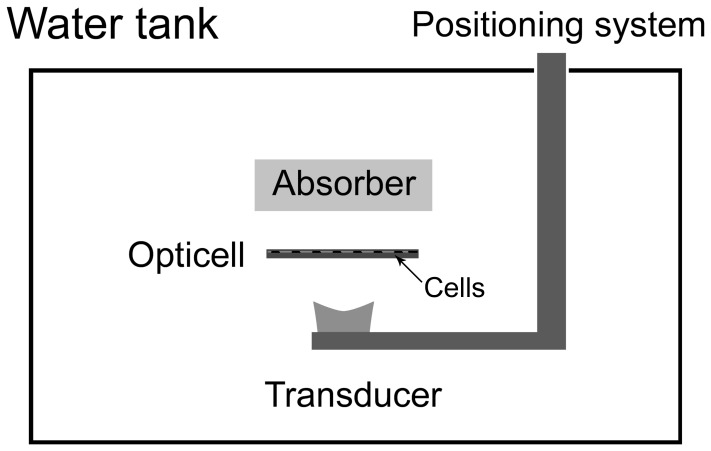
HIFU exposure setup. Our in-house built HIFU apparatus consisting of a motorized transducer, capable of movement in the x, y, and z planes, and a holder for the Opticell™ chamber submerged in a tank of degassed 37°C water. The walls of the tank and the area above the transducer and Opticell™ chamber were lined with an absorptive material, to prevent reflection of the acoustic beam and resulting standing wave formation.

The HIFU experiments were done with our in-house built excitation apparatus consisting of a custom-built ultrasound transducer that can be driven to deliver excitation at the required frequency, acoustic pressure, pulse duration and repetition. The transducer was mounted on a motorized system that allowed for precise localization and targeting of the desired area of exposure. After calibration and analysis of the pressure profile and efficiency of the transducer, we found the largest area where homogeneous pressure was obtained. This area is 6 mm in diameter and was found 10 mm before the focus of the transducer. During the characterization measurements, we also ensured that the setup for the exposure of the cells was devoid of unwanted reflections and stationary waves that could compromise the results and impair reproducibility. All experiments were conducted in a degassed water tank at 37°C with the target cells in a sealed cell culture chamber (Opticell™; Nunc, Rochester, NY, USA) with a growth area of 50 cm^2^ to which the antibody and microbubbles were added prior to HIFU exposure (Fig. 1).

### Viability and Apoptosis Assays for HIFU Parameter Optimization

Immediate cell loss caused by the stress of sonoporation alone was assessed by recording ten random images from each Opticell™ chamber, both preceding and approximately two hours following HIFU or sham treatment exposure using phase contrast microscopy. These images were then evaluated for cell count.

Decreases in viability of the remaining cells induced by the stress of sonoporation alone was assessed by trypan blue staining of cells treated with 1000 kPa or 0 kPa of pressure, in the presence of microbubbles, 24 hours after exposure to HIFU or sham treatment. Cells were trypsinized and removed from their Opticell™ chambers. The cells were then resuspended in 1×PBS, to prevent any false positives due to serum proteins in the complete medium staining blue. The cell suspension was mixed 1∶1 with 0.4% trypan blue and the numbers of stained and unstained cells were counted using the Bio-Rad TC10 Automated Cell Counter (Bio-Rad, Mississauga, ON, Canada). Ten counts were taken for each Opticell™ and then averaged. In an additional experiment, cells remaining attached 24 hours after exposure to HIFU or sham treatment were also monitored for the presence of cleaved PARP as described below in the immunofluorescence section, following fixation with 4% paraformaldehyde (PFA)-PBS for 10 minutes at room temperature.

### Molecule Transfer Using Chemical Transfection

HiPerFect (Qiagen, Toronto, ON, Canada) was used for the chemical transfer of antibodies into different cell lines. Briefly, 2.5×10^4^ cells per well were seeded onto 24-well plates and allowed to adhere. The following day, 3 µL of the transfection reagent and 4 µg/mL or 40 µg/mL of antibody were added to the adhered cells. Forty-eight hours later, cells were fixed for 10 minutes at room temperature with 4% PFA-PBS, stained using immunofluorescence and examined by microscope. Negative control wells consisted of culture medium alone or culture medium containing transfection reagents only. All chemical transfections were performed in triplicate.

### Molecule Transfer Using HIFU

For delivery of the monoclonal antibodies against tubulin and HPV16 E6, 6.0×10^5^ cells were plated as a monolayer onto one side of an Opticell™ chamber 24 hours before ultrasound exposure, according to the manufacturer’s protocol. Immediately before ultrasound exposure, the cells were washed with serum- and antibiotic-free medium and incubated with 4 µg/mL of monoclonal antibody in serum- and antibiotic-free medium for 15 minutes at 37°C with 5% CO_2_. The microbubbles were activated and applied to cells by mixing them into the culture medium right before ultrasound exposure. After exposure, the Opticell™ chambers were returned to the incubator and 2 hours later, 1.1 mL of medium was aspirated from the chamber and replaced with 1 mL FBS and 100 µL of antibiotic/antimycotic, to make the medium complete again. Forty-eight hours following ultrasound exposure, cells were fixed with 4% PFA-PBS for 10 minutes at room temperature, stained using immunofluorescence and examined by microscope.

### Immunofluorescence

Immunofluorescence was performed on fixed cells to detect the presence of tubulin or E6 antibody and to stain for p53 or cleaved PARP protein expression. For tubulin antibody detection, a secondary antibody labelled with a green-fluorescent dye, Alexa Fluor® 488 donkey anti-mouse (Life Technologies Inc., Burlington, ON, Canada), was applied at a concentration of 1∶400. The presence of E6 antibody was detected by the application of an Alexa Fluor® 594 labelled donkey anti-mouse secondary antibody (Life Technologies Inc.) applied at a concentration of 1∶800. For p53 protein staining, a monoclonal rabbit anti-human p53 protein primary antibody (Clone 318-6-11; Dako, Burlington, ON, Canada) was applied at a concentration of 1∶100, followed by an Alexa Fluor® 488 donkey anti-rabbit secondary antibody (Life Technologies Inc.), at a concentration of 1∶400. For cleaved PARP staining, a mouse monoclonal anti-human cleaved PARP primary antibody (clone 4B5BD2; Abcam, Toronto, ON, Canada) was applied at a concentration of 1∶760, followed by an Alexa Fluor® 594 labelled donkey anti-mouse secondary antibody (Life Technologies Inc.) applied at a concentration of 1∶800. Nuclear counter-staining was done with DAPI, which also allowed the nuclei to be monitored for any apoptotic changes.

### Microscopy

Live cell (phase contrast) microscopy was performed using an inverted Zeiss Axiovert 200 (Carl Zeiss Canada Ltd., North York, ON, Canada) microscope. Fixed cells were imaged with the same system, using both phase contrast and fluorescence functions. A CCD camera (Q Imaging, Surrey, BC, Canada) with 12-bit capability was used to record images. Images were taken with A-Plan 10×/0.25Ph1 and LD A-Plan 40×/0.50Ph2 objectives (Carl Zeiss Canada Ltd.) at 100- to 400-fold magnifications. An average percent of fluorescence-expressing cells was determined and used to evaluate delivery efficiency.

Alexa Fluor® fluorescence-expressing cells, as well as the total number of cells (as determined by nuclear DAPI staining) within five fields of view for every chemical transfection well or Opticell™ sonoporation treatment trial (each ∼4 cm^2^ area of Opticell™ membrane) were counted. For tubulin/E6 antibody detection and cleaved PARP staining, the number of positively expressing cells was determined visually by the authors. For p53 staining, images were digitally processed using CellProfiler software to quantify p53 fluorescence intensity and determine the percentage of p53 positive cells [42]. CellProfiler software is routinely used for both the objective quantification and subcellular localization of protein expression in the same experimentally treated cells [43], [44]. This software was used to automatically segment the nuclei in the DAPI-stained photo of the cells, using an Otsu global method with minimization of the weighted variance [45]. An image mask of each nucleus was obtained and overlaid on the corresponding photo of nuclear p53 staining of the same field of view. The intensity of p53 expression (Alexa Fluor® 488) for each nucleus was calculated as the average of green pixels inside the corresponding mask. The average and standard deviation of any green background signal in each photo was also calculated in the areas not covered by nuclei. A nucleus was considered p53 positive if the average green signal intensity of the nucleus was two times the standard deviation larger than the average green background signal intensity of the field of view. Using this software for the processing of our p53 staining was unique in that it eliminated the subjectivity of determining which cells to count as positive, which is traditionally determined visually by an individual. Since the green nuclear p53 signal is also compared relative to any background green signal for each individual photo, we also accounted for any variances in background between the images.

### Statistical Analysis

Multi-way fixed effects ANOVA was used to determine global mean differences. If differences were found, post hoc Student’s t-test or Tukey HSD analyses were performed. Interaction effects were tested in all models. Data were determined to meet parametric assumptions on the basis of independence, normality, and homogeneity of variance. Normality was tested using histogram, Q-Q plot, and Shapiro-Wilk’s test. Homogeneity of variance was tested using Bartlett’s or Levene’s test. Significance level (α) was set, *a priori*, at 0.05. Unless otherwise indicated, data are presented as means +/− SEM. R (version 2.15.0) was used for all statistical analyses.

## Results

### Optimization of HIFU Parameters

Ultrasound parameters were first optimized. Based on earlier experiments by Curiel *et al*. [46], it was determined that an acoustic pressure of 1000 kPa using a 5% duty cycle during 30 s of total exposure time (32 µs pulses at 1.5 kHz repetition rate) was optimal for molecule delivery. We used a 0.33% volume concentration of microbubbles. These parameters were employed for all subsequent experiments.

Our studies use two HPV16-positive cervical cancer-derived cell lines, SiHa and CaSki, as biological models for investigating the process of sonoporation via HIFU. SiHa contains 1 to 2 genome copies of HPV16 [47] and CaSki contains 200 to 600 genome copies of HPV16 per cell [48]. Despite genome copy number differences, there is not a direct linear relationship to the amount of expressed and functional E6 protein [49]. Adherent monolayers of these cell lines were exposed to calibrated ultrasound beams in the presence of microbubbles.

**Figure 2:**
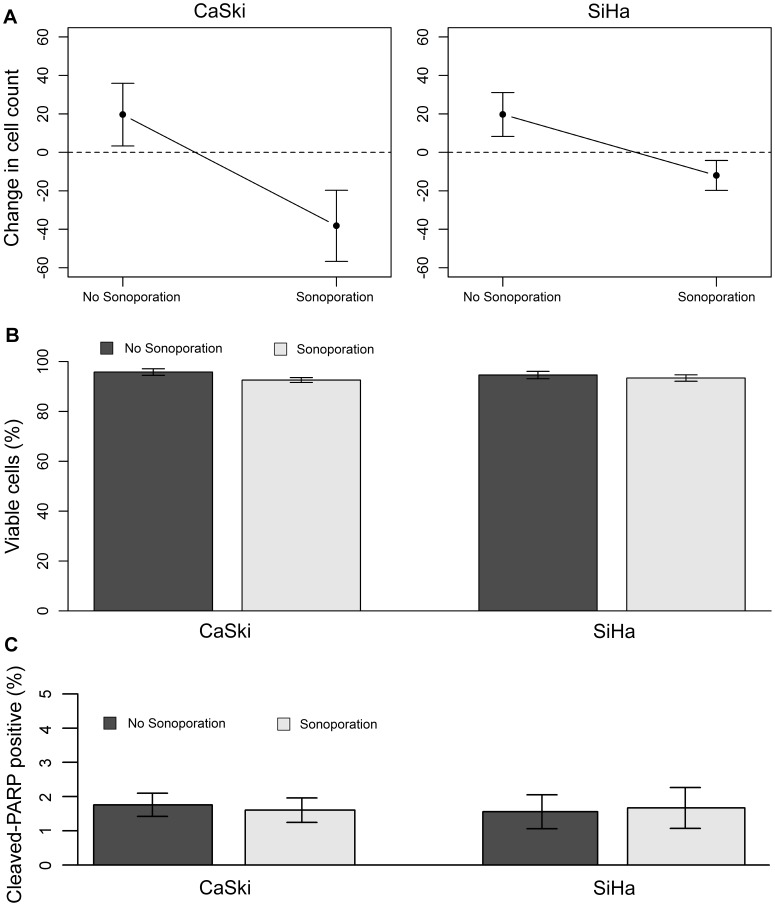
Effects of sonoporation on cell count, viability and apoptosis. (A) Change in cell count 2 hours following exposure of cells to sonoporation or sham no HIFU treatment. Sonoporation had a negative effect on cell count, (*P = *0.003, *n = *10). Statistical analysis was performed using a two-way ANOVA with Student’s two-sample t-tests post hoc. (B) Viability of remaining cells 24 hours following exposure of cells to sonoporation or sham no HIFU treatment. Sonoporation did not diminish the viability of the remaining cells (*P>*0.05, *n = *10). Statistical analysis was performed using a two-way ANOVA. (C) Percent early apoptotic cells, as evidenced by cleaved PARP staining, 24 hours following exposure of cells to sonoporation or sham no HIFU treatment. Sonoporation did not increase the number of apoptotic cells (*P>*0.05, *n* = 6), as tested by a two-way ANOVA. Error bars represent mean +/− SEM.

To quantify immediate cell loss, largely due to detachment caused by the stress of sonoporation alone under these parameters, the changes in cell count approximately 2 hours following exposure to sonoporation or no HIFU (sham) treatment were compared (Fig. 2A). Sonoporation significantly decreased cell count compared to the sham treatment, with the number of CaSki and SiHa cells decreasing by approximately 28% and 10% respectively (*P = *0.003). No corresponding detachment was seen in cells receiving the no HIFU sham treatment. In fact, slight cell growth throughout the day in the sham group was even noted. There was no significant difference between the responses of the two cell types. To monitor for further decreases in the amount of viable cells induced by the sonoporation treatment alone, the remaining attached cells were stained with trypan blue 24 hours later. Both cell types retained>90% viability, which was not significantly different from the viability of the cells in the sham treatment groups (Fig. 2B). In an additional experiment, cells remaining attached 24 hours following both treatments were also stained for cleaved PARP expression, an early marker of apoptosis. Both cell types retained <2% apoptosis, which was not significantly different from apoptosis in cells without sonoporation (Fig. 2C).

### Monoclonal Antibody Transfer with HIFU and Microbubbles is Feasible for Cervical Cancer- derived Cell Lines

**Figure 3:**
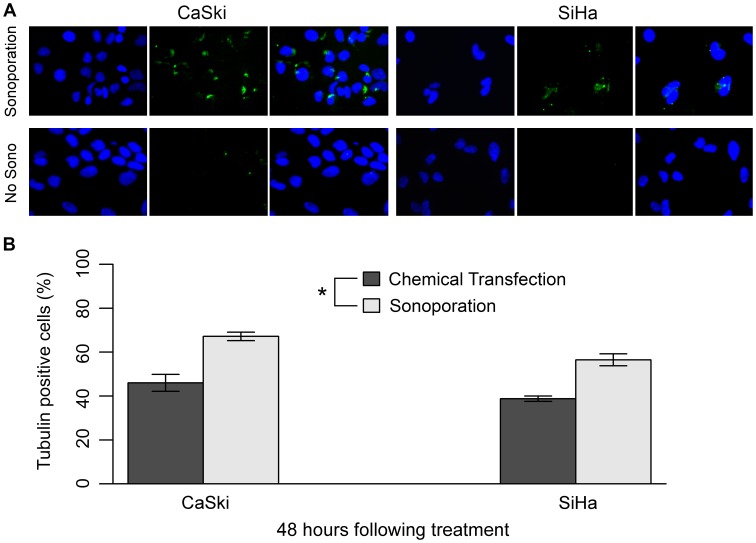
Detection of 4 µg/mL tubulin antibody within CaSki and SiHa cells. (A) Immunofluorescence detection of the tubulin antibody 48 hours following exposure of the cells to sonoporation or sham no HIFU treatment. Nuclei were counterstained blue with DAPI (left photo). Green signal indicates internalized tubulin antibody detected by the application of a secondary fluorophore (centre photo). Images merged (right photo). Cells were visualized using 400× magnification. (B) Tubulin antibody positive cells (%) 48 hours following both chemical transfection and sonoporation with 4 µg/mL antibody. Sonoporation resulted in a higher antibody transfection efficiency than that obtained with the traditional chemical reagent, HiPerFect (*P<*0.001, *n = *3). Statistical analysis was performed using a two-way ANOVA with Student’s two-sample t-tests post hoc. Error bars represent mean +/− SEM. “*” denotes significance.

We measured the effect of transfection via HIFU combined with microbubbles on the ability of CaSki and SiHa cells to take up exogenous material (i.e. cell membrane permeability). This was initially determined via a monoclonal antibody against the house-keeping gene product tubulin, using 4 µg/mL of anti-tubulin antibody, the minimum antibody concentration resulting in a detectable signal following chemical transfection and twice the minimum antibody concentration resulting in a detectable signal following sonoporation [46]. Chemical transfection data with the same antibody was then used as a baseline for comparison with our new sonoporation method. HIFU was administered in equivalent exposures over three different 4 cm^2^ zones of the Opticell™; another two zones of the same size received tubulin antibody and microbubbles, but no exposure to the ultrasound beam. Antibody detection was performed with a donkey anti-mouse secondary labelled with Alexa Fluor® 488. The tubulin antibody was detectable in approximately 56% of SiHa and 67% of CaSki cells, as evidenced by green fluorescence emission at 519 nm. Controls that omitted HIFU were all tubulin antibody-negative (Fig. 3A). When transfection with the same concentration of antibody was performed using the HiPerFect chemical reagent (Fig. 3B), we detected the tubulin antibody in approximately 39% of SiHa and 46% of CaSki cells. Statistical analysis confirms that sonoporation resulted in a significantly higher number of antibody-positive cells than chemical transfection (*P<*0.001). Interestingly, the two-way ANOVA also indicated a difference in response between the two cell types (*P = *0.009), however the post hoc Student’s two sample t-test did not conclude that CaSki had a significantly higher number of transfected cells than SiHa (*P>*0.05). This discrepancy between the ANOVA and the post hoc test for cell effect is due to a lack of resolving power associated with a low number of replicates, which can lead to a potential false-negative with the simple post hoc. Global tests such as an ANOVA are able to identify any differences with greater power, indicating that this potential difference in transfection efficiencies between CaSki and SiHa cells could be better elucidated with an experiment involving a larger number of replicates. Despite the low sample size, our data demonstrate that monoclonal antibodies can indeed be transfected into human cervical cancer-derived cell lines via sonoporation, and indicate that sonoporation results in higher transfection efficiency than that obtained with the use of traditional chemical reagents.

### Monoclonal Antibodies Against the E6 Protein Evidence the Ability to Restore the Expression of p53

We identified an optimal anti-E6 protein monoclonal antibody for future use in the sonoporation experiments, as judged by its ability to restore the expression of p53 through its targeting of E6. Once again, chemical transfection data was used to determine the baseline biological effects of the antibody, before proceeding with sonoporation experiments. Initially, a comparison of the 4C6 E6 antibody, originally developed at the University of Strasbourg [21], [50], and the F127-6G6 E6 antibody, newly developed by Arbor Vita Corporation and not previously investigated in this context, was completed using chemical transfection. The 4C6 antibody binds to an epitope in the N-terminal region of the E6 protein [50], and previous cell culture studies by Courtête *et al*. [21] have demonstrated that this antibody was able to decrease E6-mediated degradation of p53 in CaSki cells following chemical transfection. The F127-6G6 antibody’s target epitope is located near the C-terminal region of the E6 protein, though its exact binding epitope has not been fully determined yet (experiments in progress, Arbor Vita Corporation).

**Figure 4:**
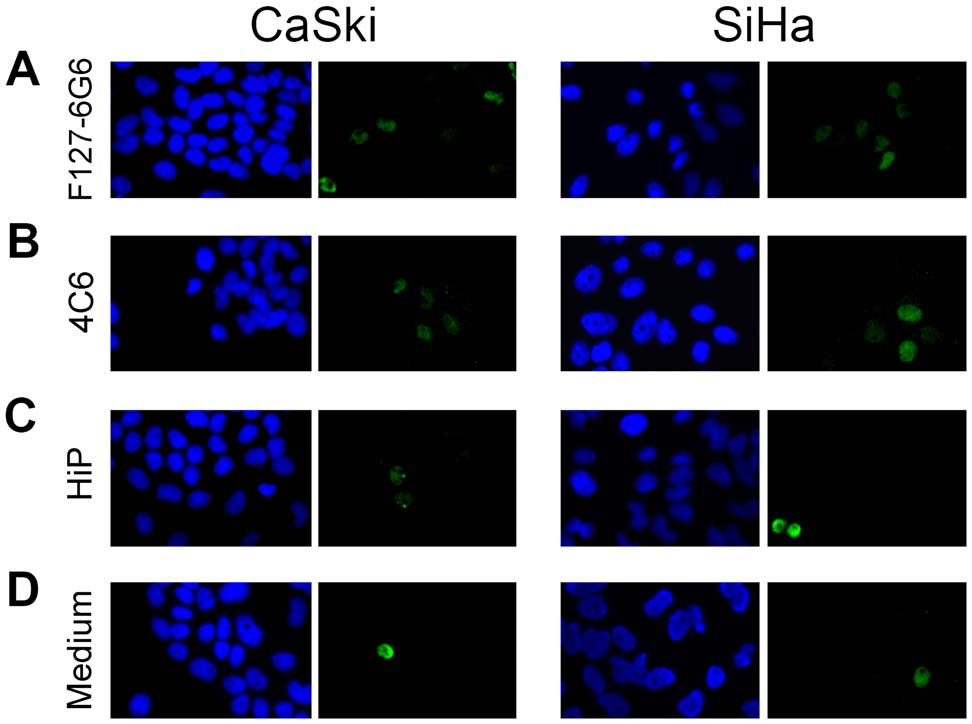
Immunofluorescence staining demonstrating p53 expression (green) following chemical transfection. (A) Transfection with 4 µg/mL F127-6G6 E6 antibody. (B) Transfection with 4 µg/mL 4C6 E6 antibody. (C) Cells treated with only the HiPerFect (HiP) transfection reagent. (D) Cells which received culture medium alone. Nuclei were counterstained blue with DAPI. Cells were visualized using 400× magnification 48 hours following transfection.

**Figure 5:**
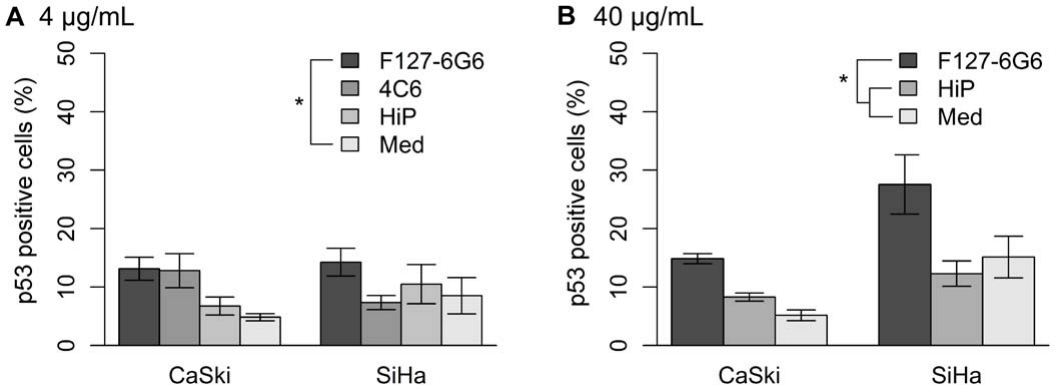
p53 expression following chemical transfection with E6 antibodies. (A) p53 positive cells (%) 48 hours following chemical transfection with 4 µg/mL of HPV16 E6 antibodies. Transfection of the F127-6G6 antibody significantly increased the number of p53 positive SiHa and CaSki cells compared to cells receiving medium alone (Med) (*P = *0.036, *n = *3) but not compared to cells treated with only the HiPerFect (HiP) transfection reagent (*P*>0.05, *n* = 3). (B) p53 positive cells (%) 48 hours following chemical transfection with 40 µg/mL of the HPV16 E6 antibody F127-6G6. Transfection of the F127-6G6 antibody significantly increased the number of p53 positive cells compared to cells treated with only the HiPerFect transfection reagent or culture medium alone (*P = *0.005, *P = *0.004 respectively, *n = *3 for both). SiHa had a significantly greater number of p53 positive cells than CaSki (*P = *0.002, *n = *3). Statistical analysis was performed using a two-way ANOVA followed by Tukey HSD contrast tests post hoc. Error bars represent mean +/− SEM. “*” denotes significance.

After 48 hours, transfection using 4 µg/mL of the anti-E6 antibody F127-6G6 into CaSki and SiHa cells resulted in a significant increase in the number of p53 positive cells per well compared to wells receiving culture medium alone (*P = *0.036), but not compared to wells treated with only the transfection reagent (Fig. 4 and 5A). Chemical transfection reagents have a cytotoxic effect on cells and this is responsible for the slight increase in p53 expression seen in the wells treated with only this reagent, which was not significantly greater than p53 expression in untreated culture medium only wells (*P>*0.05). Transfection with the anti-E6 antibody 4C6 also resulted in an increase in the number of p53 positive cells, particularly in CaSki. However, this increase was not significant. There were no significant differences in the way the p53 expression of the two cell types responded to the antibodies.

**Figure 6:**
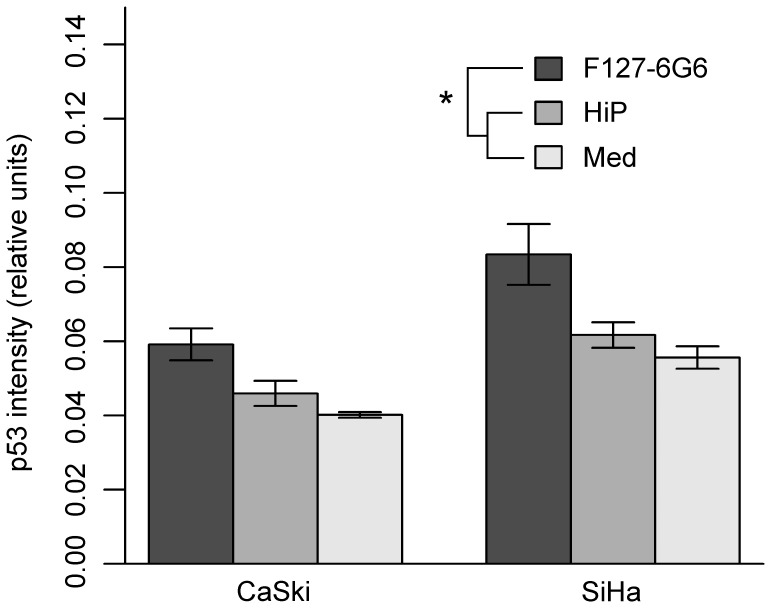
Average quantity of p53 per cell following chemical transfection with 40 µg/mL F127-6G6 E6 antibody. p53 quantity is evidenced by intensity of p53 staining. Cells transfected with the antibody show a higher quantity of p53 per cell (i.e. a higher intensity of p53 staining) after 48 hours than those in control wells treated with only the HiPerFect (HiP) transfection reagent or culture medium (Med) alone (*P* = 0.005, *P<*0.001, *n = *3 for both). Also, SiHa cells showed a greater quantity of p53 per cell than CaSki (*P<*0.001, *n = *3). Statistical analysis was performed using a two-way ANOVA followed by Tukey HSD contrast tests post hoc. Error bars represent mean +/− SEM. “*” denotes significance.

**Figure 7:**
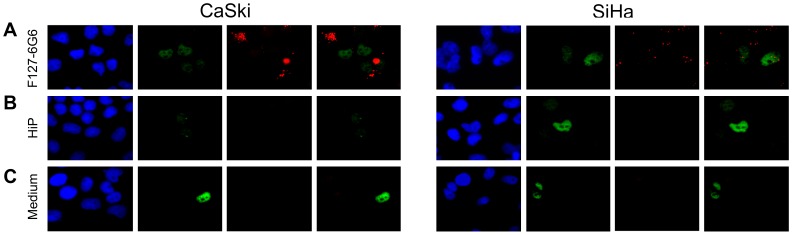
Immunofluorescence demonstrating p53 expression (green) and detection of F127-6G6 E6 antibody (red) following chemical transfection. (A) Transfection with 40 µg/mL F127-6G6 antibody. (B) Cells treated with only the HiPerFect (HiP) transfection reagent. (C) Cells receiving culture medium alone. Nuclei were counterstained blue with DAPI. p53 and F127-6G6 images merged (right most photo). Cells were visualized using 400× magnification 48 hours following transfection. The images were cropped and zoomed in to better demonstrate the colocalization of p53 and E6 antibody staining.

To further investigate the ability of the F127-6G6 antibody to restore p53 expression by preventing its E6 mediated degradation, we repeated a second chemical transfection, this time using 40 µg/mL of antibody (10× the original concentration) (Fig. 5B). For both cell types at this higher concentration, the F127-6G6 antibody resulted in a significant increase in the number of p53 positive cells compared to both wells treated with the transfection reagent alone and culture medium alone (*P = *0.005 and *P = *0.004, respectively). SiHa also had a significantly greater number of p53 positive cells than CaSki (*P = *0.002). Interestingly, at this concentration, transfection with the F127-6G6 antibody increased the average quantity of p53 per cell, as evidenced by an increase in the average intensity of the fluorescent signal coming from p53 stained cells compared to control wells (*P* = 0.005 for transfection reagent only, *P<*0.001 for medium only) (Fig. 6). SiHa cells showed a greater quantity of p53 per cell than CaSki (*P<*0.001). No significant increase in p53 staining intensity was seen at the lower F127-6G6 antibody concentration (data not shown). As with the tubulin antibody, the F127-6G6 antibody was also detectable within the cells following transfection (Fig. 7). Despite the significant increases in p53 obtained, no notable increase in the number of apoptotic cells was observed 48 hours following transfection, as evidenced by homogeneously intact nuclei.

### Cells Devoid of HPV Genetic Material do not Show Increased p53 Levels when Treated with Monoclonal Antibodies Against HPV16 E6

**Figure 8:**
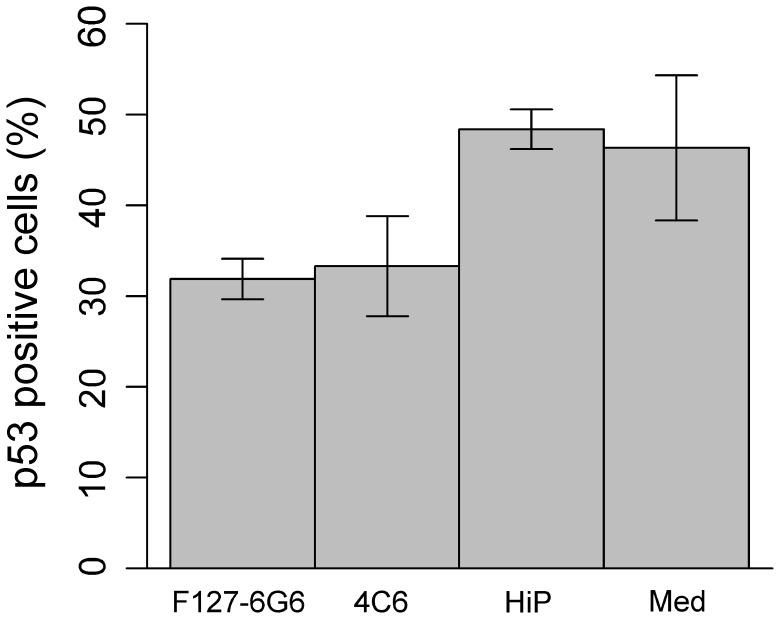
p53 positive NIKS cells (%) following chemical transfection with 4 µg/mL of HPV16 E6 antibodies. Neither antibody had an effect on the number of p53 positive NIKS cells (*P>*0.05, *n = *3) after 48 hours. (HiP) Cells treated with only the HiPerFect transfection reagent. (Med) Cells receiving culture medium alone. Statistical analysis was performed using a one-way ANOVA. Error bars represent mean +/− SEM.

To confirm the specificity of p53 augmentation in cells infected with the HPV16 virus, chemical transfection with both of the E6 antibodies was repeated using NIKS cells, which do not contain HPV genetic material. Statistical analysis confirmed that neither of the antibodies had any significant effect on p53 levels (Fig. 8). These results are to be expected given that NIKS contains no E6 oncogene. This also demonstrates that neighbouring healthy, uninfected cells in future *in vivo* models will remain unaltered by treatment with these anti-E6 molecules.

### F127-6G6 Antibody is Able Reduce E6-mediated p53 Degradation Following Sonoporation

We performed sonoporation of HPV16 positive cervical carcinoma cell lines using an E6 antibody. We monitored for increases in p53 expression which would result from the antibody entering the cell, binding to the E6 protein and preventing it from degrading p53. Since 4 µg/mL of F127-6G6 resulted in a significant increase in the number of p53 positive cells compared to control wells receiving culture medium only during the initial chemical transfection experiment and the same concentration of 4C6 did not have a significant effect, we chose to proceed with the F127-6G6 antibody. To minimize the chance of off-target cytotoxic effects, and because sonoporation resulted in a significantly better transfection efficiency than that obtained by chemical reagents, the lower 4 µg/mL concentration of F127-6G6 antibody was also used for the HIFU sonoporation experiments. A 2×2×2 factorial design was followed, to include the effects that sonoporation or antibody alone had on p53 expression and to be able to determine if there were any differences in the way that CaSki and SiHa responded to the treatment. Trials were performed in replicates of 6, with each replicate covering an area of approximately 4 cm^2^ of the Opticell™ membrane. The disruption of microbubbles was noted visually in the treatment zones after ultrasound exposure. Sonoporation experiments using 4 µg/mL of E6 antibody also confirmed that there was no effect on p53 expression in non-cancerous NIKS cells (data not shown).

**Figure 9:**
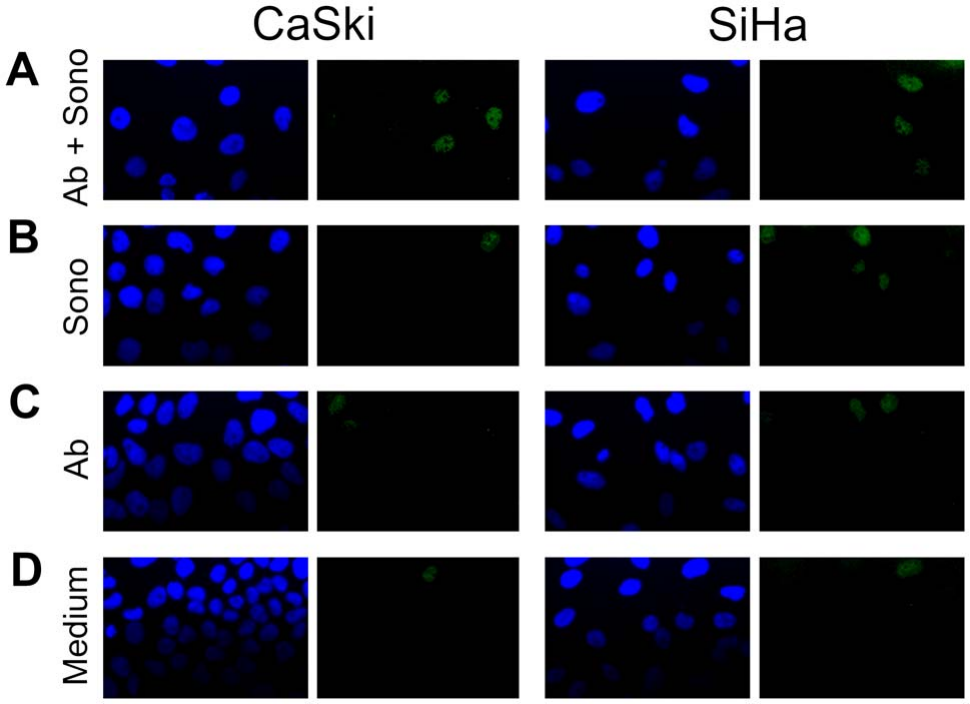
Immunofluorescence staining demonstrating p53 expression (green) following sonoporation treatment of the cells. (A) Treatment with 4 µg/mL F127-6G6 E6 antibody+sonoporation. (B) Treatment with no antibody+sonoporation. (C) Treatment with 4 µg/mL F127-6G6 E6 antibody-no sonoporation. (D) Treatment with no antibody-no sonoporation. Nuclei were counterstained blue with DAPI. Cells were visualized using 400× magnification 48 hours following sonoporation.

**Figure 10:**
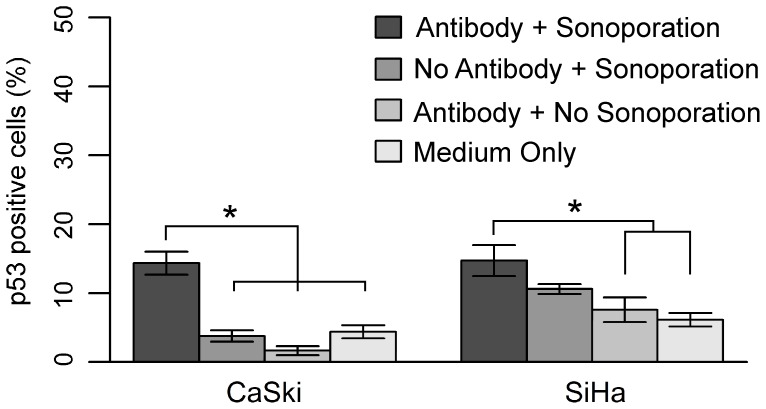
p53 positive cells (%) following the intracellular delivery of the F127-6G6 E6 antibody via sonoporation. After 48 hours, the three-way interaction between cell type, sonoporation, and antibody was significant (*P = *0.008). CaSki cells receiving 4 µg/mL of the F127-6G6 E6 antibody coupled with sonoporation (Sono) had a greater number of p53 positive cells than those in the three control groups (*P<*0.001 for all 3 comparisons, *n* = 6 for all). SiHa cells receiving 4 µg/mL of the F127-6G6 E6 antibody coupled with sonoporation had a greater number of p53 positive cells than those in the groups receiving the antibody only or no antibody and no sonoporation (*P = *0.012, *P = *0.001 respectively, *n = *6 for both). Sonoporation alone resulted in a significantly higher number of p53 positive cells in SiHa than CaSki (*P = *0.019, *n = *6). Statistical analysis was performed using a three-way ANOVA followed by Tukey HSD contrast tests post hoc. Error bars represent mean +/− SEM. “*” denotes significance.

Forty-eight hours following sonoporation, cells were stained for p53 expression (Fig. 9). The three-way interaction among cell type, sonoporation and antibody was significant (*P = *0.008) (Fig. 10). For CaSki cells, treatment with both the antibody and sonoporation resulted in significantly more p53 positive cells than controls treated with sonoporation only, antibody only, or neither (*P<*0.001 for all). All three controls were not significantly different from each other. For SiHa cells, treatment with both antibody and sonoporation resulted in significantly more p53 positive cells than the controls treated with antibody alone or no antibody and no sonoporation (*P* = 0.012, *P = *0.001 respectively), but not than the control treated with sonoporation only. Though sonoporation alone resulted in an increase in p53 positive cells compared to the other two controls in SiHa, this increase was not significant and, as seen in CaSki, none of the controls were significantly different from each other. This difference in the response of p53 expression following sonoporation alone between the two cell types was significant *(P = *0.019). One cell type did not have a significantly larger number of p53 positive cells than the other following treatment with both the antibody plus sonoporation; SiHa had a smaller magnitude of increase in p53 positive cells compared to control Opticells™ than CaSki. Though sonoporation delivery of the F127-6G6 anti-E6 antibody did increase the number of p53 positive cells, it did not notably increase the average p53 quantity (the intensity of p53 staining) per cell compared to the baseline Opticell™ which did not receive antibody or exposure to ultrasound (data not shown). Also, as was seen with the chemical transfection of F127-6G6, few apoptotic cells were noted 48 hours following transfection of this E6 antibody via sonoporation, despite the increases in p53 achieved.

## Discussion

Non-invasive, pathogen-targeted treatments belong to the new era in cancer research and may be the “magic bullet”, as originally envisioned 100 years ago by Paul Ehrlich, needed for future progress [51]. Our interdisciplinary approach allows for precise targeting of HPV16 infected cervical carcinoma cells–we can select a viral protein antibody target and guide the placement of the HIFU beam with millimetre accuracy. This type of ultrasound mechanical stimulation induces a rapid hyperpolarization of the cell membrane potential when the microbubble is in direct contact with the cell, but the potential returns to its initial value when ultrasound stimulation is stopped [52]. HIFU therefore allows temporary, localized permeabilization of the cell membrane by focusing the acoustic beam to a limited region. This approach has not been used before to deliver large molecules against HPV gene products (i.e. mRNA and proteins). Our results confirm that the chosen HIFU parameters are reasonable for our *in vitro* model, as they allow the bulk of the therapeutic effect to stem from the intracellular delivery of the cancer targeted antibody rather than sonoporation-induced cell destruction. Thus, the specific nature of our delivery system would lend easily to the *in vivo* situation where protection of neighbouring healthy, uninfected cells is critical.

This study demonstrates for the first time that sonoporation delivery of a monoclonal E6 antibody, F127-6G6, into cervical carcinoma cell lines is indeed possible and significantly increases the number of p53 positive cells. The proposed mechanism for p53 restoration is the binding of the E6 antibody to the E6 protein after it enters into the cytoplasm, which deactivates E6, preventing it from interacting with and degrading p53. It is important, however, to note that this effect on p53 was observed 48 hours following sonoporation and is likely transient, as E6 is continuously expressed.

It is interesting that sonoporation alone increased p53 expression, notably in SiHa cells. Perhaps CaSki cells, which grow in tightly packed clusters, are more resistant to the mechanical stresses caused by exposure to the ultrasound beam and microbubble cavitation. Despite this sonoporation-induced increase in p53, cell loss (both immediately and 24 hours following sonoporation) were not dramatic and should become even less of an issue in an *in vivo* situation, where the cells have surrounding tissues and structure to secure them in place. Therefore, any synergistic effects resulting from the combination of sonoporation itself and the E6 antibody should only serve to be beneficial in the selective eradication of cancerous tissue.

It is also an important finding that CaSki and SiHa cells do not respond identically to our therapy. This highlights the complexity that would be involved in the potential future treatment of tumours composed of a heterogeneous mix of cells in *in vivo* settings and further supports the need for the future development of individualized treatment plans.

Though delivery of the E6 antibody using sonoporation was effective, giving a significant restoration of p53, we were unable to induce apoptosis. This is in agreement with the findings of other groups. Courtête *et al*. [21] were unable to induce apoptosis in CaSki cells chemically transfected with the 4C6 anti-E6 antibody, though they were able to demonstrate a decrease in cellular proliferation. Thus, the anti-E6 molecule delivered inside the cell using our approach requires further optimization to improve upon this. While some E6 protein is found in the cytosol, the bulk of it is located within the nucleus [49]. In accordance with our findings, other groups have also demonstrated that sonoporation creates temporary openings in the cell membrane large enough to allow monoclonal antibodies, approximately 150 kDa, inside the cell [27], [31], [33]. However, sonoporation does not affect nuclear permeability and our F127-6G6 antibody is too large to enter the nucleus by passive diffusion through nuclear pore complexes, remaining restricted to the cytosol ([31] and references therein). High affinity antibody mimetics, such as Affibodies®, can be designed [53]. These are much smaller in size than monoclonal antibodies (only ∼6 kDa) [53] and could potentially be small enough to passively enter the nucleus, gaining access to a larger proportion of its cellular E6 target ([31] references therein), and therefore may be more successful at evoking an apoptotic response, removing the need for repetitive treatments to suppress E6 activity.

Other anti-E6 molecules, such as siRNA, may also be helpful to use in combination with our antibody approach [18]–[21]. In addition to the antibodies, sonoporation would also allow anti-E6 siRNA inside the cell. However, siRNA only needs to gain access to the perinuclear region where mRNA transcripts are located in order to function at its maximum potential. This would allow for E6 to be doubly suppressed, both at the protein level by the antibody and the transcriptional level by the siRNA. One future consideration for this combined approach is that naked siRNA is quickly degraded when delivered *in vivo*, especially during systemic injection, and may need to be packaged in nanoparticles to help protect it [54], [55]. Synergistic effects may also be obtained by combining the use of more than one E6 antibody at a time, each one targeted at a different epitope of the protein.

The main benefit of using a sonoporation delivery based anti-E6 method is its ability to be applied to *in vivo* models and clinical trials with relative ease. HIFU has become a well-established technology, not only in its applicability to many different areas of medicine, but in its potential as a useful, non-invasive treatment option. Investigations of various other applications for HIFU–including thermal drug delivery, blood brain barrier disruption, and clot thrombolysis–already regularly employ animal models [56]–[61] and some have reached the phase I/II clinical trial stage (e.g. for prostate cancer) [62]. In 2004, an MRI-guided focused ultrasound treatment for uterine fibroids even received FDA approval [63]. Our anti-E6 molecules and microbubbles can be injected intravenously, in conjunction with targeted HIFU exposure. In addition, the ultrasound beam is capable of reaching metastatic sites in deeper tissues which are not accessible by locally injected agents. Thus, the stage is set for preclinical applications of HIFU/microbubble-guided monoclonal antibody therapy of HPV-related cancers.

In summary, we have developed a credible E6 antibody delivery approach for HPV16 positive cervical carcinoma cells, using the E6 antibody, F127-6G6, in combination with HIFU and microbubbles. Our proof of concept study demonstrates a significant increase in the number of p53 positive cells occurring, as evaluated using a new, objective method for processing fluorescent microscopy images. It is necessary to completely elucidate the E6 protein epitope that the F127-6G6 antibody binds to and the mechanism by which this interferes with the E6 mediated degradation of p53. Also, the challenges related to optimizing the anti-E6 molecules delivered must still be addressed, and larger-scale studies with a greater number of replicates will be needed. The future potential available for this approach’s *in vivo* translation warrants continued study.
